# The prevalence and characteristics of frailty by frailty phenotype in rural Tanzania

**DOI:** 10.1186/s12877-018-0967-0

**Published:** 2018-11-16

**Authors:** Emma Grace Lewis, Selina Coles, Kate Howorth, John Kissima, William Gray, Sarah Urasa, Richard Walker, Catherine Dotchin

**Affiliations:** 1 Institute of Health and Society, Faculty of Medical Sciences, Newcastle University, Newcastle upon Tyne, UK; 2 Northumbria Healthcare NHS Foundation Trust, North Tyneside General Hospital, North Shields, UK; 3 The Medical school, Faculty of Medical Sciences, Newcastle University, Newcastle upon Tyne, UK; 4 Hai District Hospital, Boma Ng’ombe, Hai, Kilimanjaro, Tanzania; 5 Kilimanjaro Christian Medical Centre, Moshi, Kilimanjaro, Tanzania; 6 Education centre, North Tyneside General Hospital, Rake Lane, North Shields, NE29 8NH UK

**Keywords:** Prevalence, Frailty, Frailty phenotype, Older adults, Sub-Saharan Africa

## Abstract

**Background:**

The frailty phenotype is defined by the presence of three from the following five clinical features: weakness, slow walking speed, unintentional weight loss, exhaustion, and low physical activity. It has been widely applied in different research and clinical contexts, including across many low and middle-income countries. However, there is evidence that the operationalisation of each component of the frailty phenotype significantly alters its characteristics and predictive validity, and care is needed when applying the phenotype across settings. The study’s objective was to operationalise the frailty phenotype in a rural Tanzanian population of older community-dwelling adults.

**Methods:**

Consenting adults aged ≥60 years, and resident in five randomly selected villages of Hai district Demographic Surveillance Site, were eligible to participate in this cross-sectional study. From a screened sample of 1207 older adults, 235 were randomised and consented to an assessment of their frailty status by the frailty phenotype. Trained research fieldworkers (Tanzanian medical doctors and nurses) carried out measurements and questionnaires at local village centres or at participants’ homes.

**Results:**

The prevalence of the frailty phenotype, calculated from complete data for 196 participants, was 9.25% (95% CI 4.39–14.12) When missing data were counted as meeting frailty criterion (i.e. missing due to inability to perform an assessment), the prevalence increased to 11.22% (95% CI 7.11–15.32). Frailty by phenotype criteria was more common in older age groups, and was associated with self-assessed poor health and depression symptoms.

**Conclusions:**

Frailty can be successfully estimated using the frailty phenotype, however there are challenges in its operationalisation cross-culturally. Further work is needed to explore the potential clinical application of the frailty phenotype in such settings.

**Electronic supplementary material:**

The online version of this article (10.1186/s12877-018-0967-0) contains supplementary material, which is available to authorized users.

## Background

Frailty was described in 2001, as being a syndromic condition, distinct from, but overlapping with, disability and comorbidity [1]. The syndromic approach to frailty asserts that frailty can be described through the measurement of five physical features, and that frailty is present when three of these features are present, and pre-frailty when 1–2 features are present. The five physical features are: unintentional weight loss, weakness (low hand grip-strength), exhaustion, slow walking speed and low physical activity [1, 2]. The phenotype model is based on a proposed theoretical “cycle of frailty” which hypothesises that clinical features measurable in frailty, and its reduced physiologic reserve, are due to a cycle of factors including disease, chronic undernutrition, sarcopenia, and a reduced resting metabolic rate [1, 3]. Since the original Frailty Phenotype (FP) described by Fried et al. as part of the Cardiovascular Health Study [1], the phenotype model has been adapted and operationalised according to differing study designs, methodologies and settings.

In low and middle-income countries (LMIC), a recent systematic review found thirty six studies had used the FP to research frailty [4]. The need for cultural adaption of the phenotype model in LMIC settings, such as Tanzania, is particularly important due to issues such as resource constraints, lower background education levels and societal health and functional norms. How the FP is operationalised impacts on the prevalence of frailty, and its predictive ability [5]. A recent study has demonstrated this phenomenon when estimating the prevalence of frailty among older adults in rural Agincourt, South Africa, where each of the nine iterations of the FP constructed, gave corresponding estimates of prevalence varying between 3.0 and 13.2% [6]. However, the benefits of the FP include its relative ease of application, making it a potential tool for screening. The FP could be applied prior to any clinical assessment serving to highlight major problems or identify those most in need [7, 8]. Another benefit to the phenotype approach is the minimal amount of data required for its calculation, particularly when compared with the deficit accumulation approach to frailty (frailty index), which requires a minimum of 20 deficits to be counted in order to produce stable estimates [9, 10]. Therefore, the study’s aim was to operationalise and measure the prevalence of the FP in a rural Tanzanian population of older community-dwelling adults.

## Methods

A total sample of 1207 adults, living in five randomly selected villages in Hai district Demographic Surveillance Site (DSS), and aged ≥60 years were screened using the Brief Frailty Instrument for Tanzania (B-FIT) tool [11]. This short questionnaire, previously developed by our research team, was used to categorise participants into non-frail, pre-frail or frail [11]. A random selection of 79 (8.9%) of non-frail, 120 (42.1%) of pre-frail and 37 (94.7%) of frail formed a frailty-weighted cohort of 236 who underwent Comprehensive Geriatric Assessments (CGA), following the recommendations of the British Geriatrics Society [12]. One case was excluded due to large amounts of missing data, leaving 235 older adults, who were assessed for frailty according to the FP, in their homes or at a convenient local centre. Our construction of the Hai district DSS FP, is compared with Fried’s FP in Table 1. In constructing a FP in our setting, we aimed to keep as close as possible to Fried’s FP, so the five measured components of frailty were operationalised thus:

- *Weakness*: Hand grip strength (HGS) was measured with participants sitting upright with the arm in flexion at 90 degrees. Three measurements were averaged from the participant’s dominant hand using a JAMAR Hydraulic Hand Dynamometer (Model J000105, Lafayette Instruments, Lafayette, IN, USA).
- *Walking speed*: Participants were asked to walk in a straight line, a distance of 4.5 m (15 ft). The distance was measured out on a flat floor surface using a rigid tape measure. The type of floor surface varied, for example participants who were assessed at their homes were often assessed walking outside due to limited space indoors. However, if the surface was slippery due to recent rain, or uneven, a surface indoors was found. At the local assessment centres (church buildings, dispensaries and local schools) smooth concrete floors were available. Walking speed was not adjusted for height or sex. Participants walked in their usual footwear and were permitted to use any walking aids.
- *Exhaustion*: A positive response was counted, to either of the two Centre for Epidemiological Studies Depression scale (CES-D) statements [13]: “I felt that everything I did was an effort” or “I could not get going”. These questions were translated verbatim to Swahili, and participants were asked to grade how often in the past week they had felt this way.
- *Weight loss*: Participants were asked “Have you lost weight during the last 3 months?” with the option of answering “Weight loss greater than 3kg”, “weight loss between 1 and 3kg”, “no weight loss” or “does not know”. This variable was turned from a categorical to a binary variable for analysis as described in Table 1. BMI was calculated from weight measured using Microlife Diagnostic WS 80 digital weighing scales and height using the Marsden Leicester Height Measure. Given that no serial measurements were possible, self-reported weight loss was preferred as a measure of unintentional weight loss.
- *Low physical activity*: The International Physical Activity Questionnaire (IPAQ), [14] was used to record participants’ estimations of their physical activity over the preceding 7 days. The IPAQ has been used widely, and in similar studies of older adults, for example the Ibadan study of ageing, from Nigeria [15]. Those who reported not being able to carry out tasks of moderate physical activity on any day of the previous week scored positively on this parameter. This was felt to be appropriate, given that carrying out “small works” in the home (such as using a winnowing basket or sharpening knives) was identified as a common norm for older adults in this context when developing a culturally adapted instrumental activities of daily living (IADLs) screening tool [16].

**Table 1:** Comparing operationalisation of the Hai DSS frailty phenotype with fried’s frailty phenotype

Component measured in both	Hai DSS frailty phenotype	Fried’s frailty phenotype
Weakness (low HGS): Average of three measurements in the dominant hand using the JAMAR hand-held dynamometer.	Frail criterion met if average HGS in the dominant hand <21Kg in males or < 10Kg in females based on the median HGS in African adults aged 61–70: 18 (10–25) women, 30 (21–38) men [21].	Stratified by gender and body mass index (BMI) quartiles: Men Cut-off for grip strength (Kg) criterion for frailty BMI ≤ 24 ≤ 29 BMI 24.1–26 ≤ 30 BMI 26.1–28 ≤ 30 BMI > 28 ≤ 32 Women BMI ≤ 23 ≤ 17 BMI 23.1–26 ≤ 17.3 BMI 26.1–29 ≤ 18 BMI > 29 ≤ 21
Slow walking speed: Walking at usual pace, 4.5 m (15 ft) use of walking aid is permitted.	The slowest quintile of the sample’s walking speed was taken as cut-off: ≥11.12 s to walk 4.5 m distance (not stratified by gender or height).	Slowest quintile stratified by gender and height Men Height ≤ 173 cm ≥7 s Height > 173 cm ≥6 s Women Height ≤ 159 cm ≥7 s Height > 159 cm ≥6 s
Self-reported exhaustion: CES–D Depression Scale questions were used. Frailty criterion if either was felt to be present a “moderate amount of the time” or “most of the time” over the past week.	The CES-D questions were verbatim translated into Swahili by a Tanzanian doctor and statements read aloud and scored in the same manner as the Fried FP.	CES–D Depression Scale questions, the following two statements are read. (a) I felt that everything I did was an effort; (b) I could not get going. Frailty criterion if either was felt to be present a “moderate amount of the time” or “most of the time” over the past week.
Unintentional weight loss:	Has the participant/older person lost weight during the last 3 months? Frailty criterion were met if the participant answered either “yes, more than 3 kg”, or “yes, between 1 and 3 kg”.	“In the last year, have you lost more than 10 pounds unintentionally (i.e., not due to dieting or exercise)?” If yes, then frail for weight loss criterion, OR ≥5% of body weight lost in the past year unintentionally.
Low physical activity	Taken from the IPAQ: “During the last 7 days, on how many days did you do moderate physical activities like gardening, cleaning, bicycling at a regular pace, swimming or other fitness activities?” Those who answered “0”, were categorised as meeting frailty criterion.	Based on the short version of the Minnesota Leisure Time Activity questionnaire Kcals per week expended are calculated using standardized algorithm. This variable is stratified by gender, with frailty criterion met if less than the lowest quintile of energy expended. *Men*: < 383 Kcals/week *Women*: < 270 Kcals/week.

### Frailty phenotype

Other recorded parameters: A questionnaire was conducted asking participants or responding close relatives about socio-demographic characteristics as well as self-reported diagnoses, including HIV-infection. The IDEA cognitive [17], and EURO-D [18], screening tools were used to screen for cognitive function and depression respectively. A short culturally specific Instrumental Activities of Daily Living (IADL) tool was used [16], where difficulty with any one of eleven instrumental activities, (e.g. carrying out small works in the home, giving advice and presiding over ceremonies) was classified as IADL disability.

### Setting

The study was carried out across five villages in Hai DSS of rural Northern Tanzania between 24th February and 9th of August 2017. The five villages were stratified according to high-, middle-, and low-land locations, due to differing climate, ethnic background and agricultural practices across these strata.

The majority of the background population were subsistence farmers reliant on non-mechanised farming practises and on seasonal rains. There are two rainy seasons per year in this region, the long rains of February–April and short rains of November–December. The short rains which were expected in November 2016 were disappointing, meaning that the second harvest of the year was poor. The data collection period took place during the long rainy season of 2017, and into the first harvest season.

### Data analysis

Data were collected by hand-held tablet devices using Open Data Kit (ODK) open source software [19]. Completed forms were uploaded to a secure ODK 0.2 aggregate online database. Downloaded data were cleaned in Microsoft Excel 2016 workbooks and imported to Stata/SE 15.0 for analysis. Simple descriptive statistics were calculated and unadjusted Chi-squared values are presented for frailty by variables of interest (Table 3). The alpha value was set at 0.05. When calculating the prevalence of frailty, the weighted stratification was taken into account, using inverse proportions. To calculate confidence intervals (CI), bootstrapping (Stata command ‘svyset’) was used to control for clustering by village and to adjust for the stratified weighting [12]. In primary analysis, cases with missing data necessary for calculating the FP were excluded from the analysis, and thus, were assumed missing completely at random and non-informative. In secondary analysis, values for missing data were imputed under the assumption that they were not missing completely at random and informative.

## Results

### Prevalence of frailty

The total study cohort of 235 people comprised 136 females (57.9%) and the average age was 74.8 years. The prevalence of frailty by Hai DSS FP, calculated from complete data for 196 participants, was 9.25% (95% CI 4.39–14.12).

### Impact of missing data on estimated frailty prevalence

The number of assessments of walking speed and grip strength which were missing were 37 and 18, respectively. Interestingly, 11 of the 37 missing walking speed assessments were due to immobility following a stroke, suggesting that such data were not missing completely at random, and that these data were informative. Overall, of the 39 participants missing data for calculation of their FP, 36 (92.3%) were deemed frail by CGA [12]. In secondary analyses, except for two erroneous walking speed recordings which were deleted, all other missing data were presumed missing due to being unable to complete the assessment (i.e. due to frailty rather than missing at random), and thus categorised as meeting the frailty criterion. The CGA-derived frailty diagnoses for participants with missing data have been provided as supplementary online data in Additional file 1: Table S1. When the Hai DSS FP was calculated using this approach the prevalence of frailty increased to 11.22% (95% CI 7.11–15.32).

### Correlates of frailty by phenotype

The mean walking speed was 0.65 m/s (std. dev 0.40, range 0.04–4.29 m/s), 0.72 m/s in men and 0.60 m/s in women. The mean dominant HGS was 21.9Kg (Std. dev 8.5, range 4.3–51.3), 18.92 Kg in women and 26.02 Kg in men. The mean BMI in women was 25.01 (std. dev 5.2, range 13.9–47.2), and in men was 21.21 (std. dev 5.5, range 13.4–53.3). Table 2 reports the frequencies and percentages of frailty components by sex. Around one third of participants had no frailty components (*n* = 63, 32.1%), while roughly one third had one frailty component (*n* = 67, 34.2%), and two participants fulfilled all five components of the FP criteria.

**Table 2:** The Frequencies and percentages of Hai DSS frailty phenotype components

Frequency (%) of frailty components	Total *n* = 196	Males *n* = 82	Females *n* = 114
Exhaustion	82 (41.82)	32 (39.02)	50 (43.86)
Weight loss	53 (27.04)	23 (28.05)	30 (26.32)
Low physical activity	48 (24.49)	15 (18.29)	33 (28.95)
Slow walking	39 (19.90)	10 (12.20)	29 (25.44)
Weakness (Hand-grip strength)	29 (14.80)	22 (26.83)	7 (6.14)
Number of frailty components			
0	63 (32.14)	31 (37.80)	32 (28.07)
1	67 (34.18)	23 (28.05)	44 (38.60)
2	32 (16.33)	15 (18.29)	17 (14.91)
3	18 (9.18)	4 (4.88)	14 (12.28)
4	14 (7.14)	8 (9.76)	6 (5.26)
5	2 (1.02)	1 (1.22)	1 (0.88)

Frailty by Hai DSS FP was associated with older age groups, self-assessed poor health, self-reported chronic diseases and with self-assessed frailty status (Table 3). It is important to note that four individuals from 196, disclosed their HIV-infected status. Being assessed at home, rather than at a local centre was strongly associated with frailty by univariate analysis. Almost half of participants were married (*n* = 97, 49.5%), however frailty was significantly associated with being widowed, separated/divorced or single. Low levels of schooling, and low literacy levels, were also significant associations with frailty with 53 participants, (27.0%) having received no formal education. High numbers considered themselves to be ill (*n* = 119, 60.7%), or living with frailty (*n* = 116, 59.1%). Depression symptoms, as reported by the EURO-D screening tool [18], were significantly associated with frailty, as was a score indicative of cognitive impairment, according to the IDEA cognitive screening tool [17]. Around a fifth of participants were found to be underweight, according to BMI, while almost two fifths were either overweight or obese (Table 3), yet BMI was not found to be associated with frailty on univariate analysis.

**Table 3:** Demographic and clinical characteristics of the study sample according to Hai DSS frailty phenotype status

Demographic characteristic	Total sample *n* = 196	FP not-frail *n* = 63	FP pre-frail *n* = 99	FP frail *n* = 34	Pearson Chi^2^ (*P* value) for FP frailty vs FP not-frail/FP pre-frail
Age group					
60–69	85	37 (43.53)	43 (50.59)	5 (5.88)	
70–79	61	20 (32.79)	31 (50.82)	10 (16.39)	
≥80	50	6 (12.00)	25 (50.00)	19 (38.00)	**22.705 (< 0.0001)**
Sex					
Male	82	31 (37.80)	38 (46.34)	13 (15.85)	
Female	114	32 (28.07)	61 (53.51)	21 (18.42)	0.219 (0.640)
Location where assessed					
Elsewhere	139	52 (37.41)	76 (54.68)	11 (7.91)	
Home	57	11 (19.30)	23 (40.35)	23 (40.35)	**29.664 (< 0.0001)**
Tribe					
Mchagga	176	59 (33.52)	88 (50.00)	29 (16.48)	
Others	20	4 (20.00)	11 (55.00)	5 (25.00)	0.909 (0.340)
Living arrangements					
Lives with others	174	56 (32.18)	86 (49.43)	32 (18.39)	
Lives alone	21	7 (33.33)	12 (57.14)	2 (9.52)	1.023 (0.312)
Marital status					
Married	97	39 (40.21)	48 (49.48)	10 (10.31)	
Widowed, Separated/divorced or Single	99	24 (24.24)	51 (50.52)	24 (24.24)	**6.633 (0.010)**
Education					
Secondary and higher education	16	11 (68.75)	3 (18.75)	2 (12.50)	
Primary completed	56	21 (37.50)	31 (55.36)	4 (7.14)	
Some primary	71	21 (29.58)	37 (52.11)	13 (18.31)	
No school	53	10 (18.87)	28 (52.83)	15 (28.30)	**8.811 (0.032)**
Literacy “Do you know how to read and write? (Yes if able/was able to read or write)”					
Able to read/write well	89	41 (46.07)	39 (43.82)	9 (10.11)	
Read/write with difficulty	52	13 (25.00)	30 (57.69)	9 (17.31)	
Unable to read/write	55	9 (16.36)	30 (54.55)	16 (29.09)	**8.539 (0.014)**
EURO-D score					
Depression symptoms ≤3/12	105	43 (40.95)	49 (46.67)	13 (12.38)	
Depression symptoms ≥4/12	91	20 (21.98)	50 (54.95)	21 (23.38)	**3.889 (0.049)**
IDEA cognitive test score					
0–4 (poor cognitive function)	20	2 (10.00)	9 (45.00)	9 (45.00)	
5–7 (moderate cognitive function)	37	6 (16.22)	22 (59.46)	9 (24.32)	
8–12 (good cognitive function)	139	55 (39.57)	68 (48.92)	16 (11.51)	**15.225 (< 0.0001)**
Functional ability					
≥1 ADL disability	47	5 (10.64)	20 (42.55)	22 (46.81)	**37.428 (< 0.0001)**
≥1 IADL disability	60	8 (13.33)	24 (40.00)	28 (46.67)	**51.845 (< 0.0001)**
Healthcare provision					
Health insurance	51	19 (37.25)	22 (43.14)	10 (19.61)	
No health insurance	144	43 (29.86)	77 (53.47)	24 (16.67)	0.226 (0.634)
Ability to earn money or produce food					
Working	59	27 (45.76)	31 (52.54)	1 (1.69)	
Not currently working	137	36 (26.28)	68 (49.64)	33 (24.09)	**14.422 (< 0.0001)**
Household facilities					
Electricity	89	31 (34.83)	38 (42.70)	20 (22.47)	
No electricity	107	32 (29.91)	61 (57.01)	14 (13.08)	2.986 (0.084)
Provision for old age					
Pension	14	8 (57.14)	5 (35.71)	1 (7.14)	
No pension	182	55 (30.22)	94 (51.65)	33 (18.13)	1.094 (0.295)
“Do you consider yourself currently ill?”					
Yes (Ill)	119	17 (14.29)	69 (57.98)	33 (27.73)	
No (not ill)	77	46 (59.74)	30 (38.96)	1 (1.30)	**22.781 (< 0.0001)**
“Do you consider yourself to be living with frailty currently?”					
Yes (Frail)	116	22 (18.97)	65 (56.03)	29 (25.00)	
No (not frail)	80	41 (51.25)	34 (42.50)	5 (6.25)	**11.609 (0.001)**
Self-assessed health: “How is your health?”					
Good/Very good	42	24 (57.14)	16 (38.10)	2 (4.76)	
Neither good nor poor	99	35 (35.35)	51 (51.52)	13 (13.13)	
Poor/Very poor	54	4 (7.41)	31 (57.41)	19 (35.19)	**17.778 (< 0.0001)**
Self-reported medical diagnoses: “Have you ever been told you have a diagnosis of any of the following?”					
Diabetes	19	7 (36.84)	8 (42.11)	4 (21.05)	0.201 (0.654)
Hypertension	58	18 (31.03)	27 (46.55)	13 (22.41)	1.475 (0.225)
Cataracts	15	3 (20.00)	6 (40.00)	6 (40.00)	**5.747 (0.017)**
Stroke	8	1 (12.50)	3 (37.50)	4 (50.00)	**6.145 (0.013)**
Heart disease	11	4 (36.36)	4 (36.36)	3 (27.27)	0.800 (0.371)
Arthritis	44	7 (15.91)	24 (54.55)	13 (29.55)	**5.788 (0.016)**
Depression	12	2 (16.67)	8 (66.67)	2 (16.67)	0.004 (0.949)
Chronic respiratory condition (asthma/COPD)	13	4 (30.77)	6 (46.15)	3 (23.08)	0.318 (0.572)
Number of self-reported chronic diseases					
0	70	27 (38.57)	40 (57.14)	3 (4.29)	
1	67	24 (35.82)	30 (44.78)	13 (19.40)	
≥2	59	12 (20.34)	29 (49.15)	18 (30.51)	**15.654 (< 0.0001)**
BMI kg/m^2^					
Underweight (< 18.49)	40	11 (27.50)	24 (60.00)	5 (12.50)	
Normal weight (18.5–24.99)	79	21 (26.58)	41 (51.90)	17 (21.52)	
Overweight (25.00–29.99)	54	22 (40.74)	24 (44.44)	8 (14.81)	
Obese (≥30.00)	21	9 (42.86)	9 (42.86)	3 (14.29)	2.008 (0.571)

Pearson Chi^2^ and *P* values in bold were significant (significance level α = 0.05)

The number of chronic diseases was derived from self-reported diagnoses of any of the following; (diabetes, hypertension, stroke, cataracts, arthritis, heart disease, respiratory disease, HIV, TB, anaemia, depression, dementia, other mental health conditions, gastro-intestinal conditions, prostatic/urinary conditions, renal failure, or cancer)

**Fig. 1:**
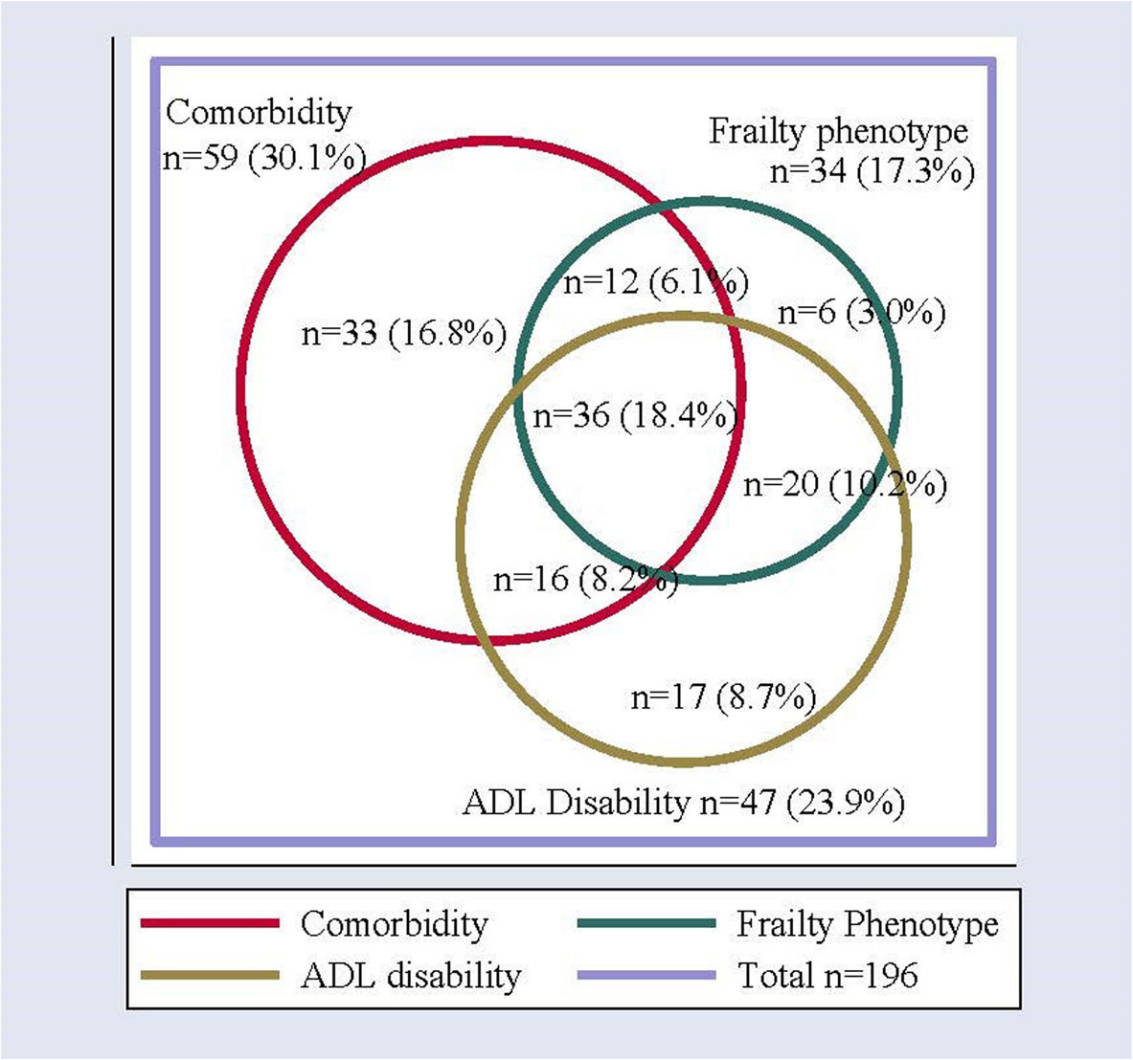
Proportional Venn Diagram Illustrating the Frequencies and Percentages of Comorbidity, Disability and Frailty by Hai DSS Frailty Phenotype. Legend: Frequencies and percentages from the total sample (*n* = 196) are described. Comorbidity *n* = 59, was categorised as anyone self-reporting ≥2 diagnoses from the following (diabetes, hypertension, stroke, cataracts, arthritis, heart disease, respiratory disease, HIV, TB, anaemia, depression, dementia, other mental health conditions, gastro-intestinal conditions, prostatic/urinary conditions, renal failure, or cancer). ADL Disability *n* = 47 was categorised as being unable to do any of the Barthel Index [37] activities of daily living

Frailty, comorbidity and ADL disability were found to be distinct, but overlapping entities. The frequencies and percentages are shown in Fig. 1. Notably, almost a fifth overlapped in having all three (*n* = 36, 18.4%). Twenty (10.2%) overlapped in having both disability and frailty, while 12 (6.1%) overlapped in having dual diagnoses of comorbidity and frailty.

## Discussion

### The prevalence of frailty by frailty phenotype

The prevalence of frailty according to the Hai DSS FP is lower than was found by the CGA, which found a frailty prevalence of 19.1% within the same study population [12]. One major reason for this is the fact that the FP requires participants to have a certain level of functioning in order to be assessed. They should be mobile and able to walk (walking aids are permitted), they should be able to comprehend and follow commands well enough to undertake HGS testing. The majority of missing data for assessment of FP were from patients who were immobile and/or cognitively impaired, for example due to stroke or dementia [12].

In the original Fried paper, older adults with certain clinical characteristics were excluded because their diagnoses may confound the results, by mimicking frailty [1]. For example, those with a history of Parkinson’s disease, stroke, and poor cognitive function. Those taking medications for depression, Parkinson’s disease or dementia were also excluded. In our rural sub-Saharan Africa (SSA) setting, excluding these participants was not possible, due to low levels of formal diagnosis, limited medication availability and poor access to healthcare. Additionally, we feel that their exclusion may significantly under-represent the true burden of frailty. This is important, given that in Europe and South Africa it has been shown that older community-dwelling adults who could not be assessed fully using the FP had higher mortality rates than those who could be assessed [6, 20]. Longitudinal assessment of this cohort would reveal whether this finding is the case in our setting.

### Weakness by hand grip strength

HGS varies by ethnicity and world region, thus we have appropriately adjusted our normal values in keeping with the most up-to-date values for HGS norms in older Africans [21]. A comprehensive review of normative HGS values found that developing world regions had significantly lower HGS compared with developed regions [22], and taking a life-course approach to ageing, postulate that factors such as early growth and nutrition may be important factors accounting for this disparity. The difference in grip strength (of 14.7 Kg in males and 5.7 Kg in females) between a rural older Ghanaian study population and a comparative Dutch population disappeared when the authors standardised the Ghanaian study population against the age- group and sex- specific height and BMI of the Dutch study population [23]. Therefore, regional and ethnic variations found in HGS may, to some extent be accounted for by height and BMI [23]. The most important determinants of HGS variation globally is a complex question. Undoubtedly genetic factors contribute, however there is an increasing body of evidence for the influence of environmental factors such as early life socio-economic status on HGS variation in old age [24].

The cycle of frailty hypothesis, states that chronic undernutrition (protein, energy and micronutrient deficiencies) is integral in the development and progression of frailty [1, 3]. Indeed, dietary protein intake has been shown to be protective against loss of HGS [25]. We hypothesise that nutritional factors, including a low protein and low micronutrient diet, may be important in the development of frailty in rural SSA, supporting findings from Malawi [26]. However, more detailed assessments of dietary intake and outcomes are required in order to make comparisons with older adults in high income country (HIC) settings.

### Slow walking speed

Given that there are no published norms of walking speed across SSA, we opted to use the slowest quintile as a cut-off. Stratifying by sex and height did not significantly influence our findings, but added to the complexity. The WHO Study of AGEing and Adult Health (SAGE) found a range of walking speeds between the slowest, (Russia at 0.61 m/s) and the fastest, (China, at 0.88 m/s) [27]. Our mean walking speed was slow, (0.65 m/s) when compared with the SAGE countries, and with walking speeds of HICs. For example a meta-analysis of nine cohort studies predicted a life expectancy above the median for older adults walking faster than 0.8 m/s [28]. As discussed in the methods, participants’ footwear, unadjusted visual impairments, uneven ground surfaces and lack of appropriate walking aids may have led to slower walking speeds.

### The concept of exhaustion

Exhaustion is a subjective and culturally dependent variable. In order to remain as close as possible to the original Fried FP, the same questions taken from the CES-D depression screening questionnaire were used, with a direct translation into Swahili. However, qualitative exploration of the meaning of “exhaustion” is needed, to ensure that this is a valid measure in this setting. For example the CES-D question; “How often in the last week did you feel you could not get going?” could have been interpreted literally as not having access to a means of transport. The fact that the majority of our study participants self-identified as living with frailty (see Table 3), is interesting in itself, suggesting that the translated term for frailty; *“udhaifu wa wazee”* or literally “weakness of the older people” doesn’t hold the same negative connotations as it does in “westernised” cultures [29]. The Agincourt frailty study faced similar translation and interpretation challenges in using the CES-D questions, and substituted a question on self-reported health status [6].

### Unintentional weight loss

We recorded any recent self-reported weight loss as being significant, and presumed unintentional. In the context of the HIV/AIDS epidemic, weight loss and a slim body habitus may be associated with HIV [30], which may have influenced our respondents’ willingness to admit to weight loss, given that it might imply infection. Cultural views on body habitus are often counter to “western” ideas, whereby being overweight may be associated with health, wealth and social status [31]. Indeed, it would be very unusual for someone in this setting to lose weight intentionally. Given that subsistence farming is the main source of food and household income, the fact that the last short rainy season had led to a poor harvest may have influenced some of the survey responses, particularly in relation to food intake and weight loss. Very few studies of nutritional status of older adults in SSA have been conducted rurally and very few are longitudinal [32]. However, in rural Gabon a cross-sectional study showed a 25.6% prevalence of underweight by BMI among older adults, which increased to 28.2% in the rainy season [33]. In our sample the mean BMI was 25.0 for women and 21.2 for men, with 20.4% (*n* = 40) categorised as underweight. Despite a global increase in obesity it may be that in such settings, older people are at an increased risk from undernutrition, due to food and income insecurity. A cross-sectional study conducted in urban Kenya, Uganda and Tanzania described the majority of older adults as being “nutritionally vulnerable” due to problems accessing food [34]. As a consequence, the relationship between frailty and chronic undernutrition is likely to be stronger in rural SSA than might be seen in HICs and warrants further investigation.

### Low physical activity

We took inability to conduct moderately difficult physical activity on any day of the week as a criterion marker for frailty. Strengths of this method are that older adults in this setting are frequently expected to take part in activities such as sweeping and gardening on a daily basis [16]. However, the measure is confounded by cognitive impairment, in a similar manner to the Minnesotta Leisure Time Physical Activity Questionnaire, in that activities such as tennis require high levels of cognitive functioning, as are required for common IADLs [8]. Given that we did not exclude older adults with cognitive impairment from our study sample, there is the possibility that this component may have been measuring their ability to perform IADLs, as assessed by the IDEA-IADL questionnaire [16]. Taking a broader multidimensional approach to frailty assessment however, this is academic, if what matters is the older adult’s functional capacity.

### Study limitations

The two parameters where these data’s objectivity could be improved are in the measurement of weight loss and calculation of low energy expenditure. An objective measurement of weight loss would have been beneficial in providing a more accurate estimate of this component of the Hai DSS FP, but would have involved repeated home visits to remote rural areas across difficult terrain, particularly in the rainy season. One main weakness of this study is that we were unable to measure an estimated energy expenditure based on data from the IPAQ due to technical difficulties recording these data. However, the IPAQ is thought to have better reliability in urban as compared to rural samples, and it may have limited validity in rural and low literacy settings such as ours [14]. There is a need for more research into the physical activity of older adults in rural SSA (e.g. using accelerometers) in order to better assess low physical activity in this setting, where it is presumed that older adults remain more physically active than might be seen in HICs, where retirement from work is the norm.

More data on the dietary intake of older adults in this setting would help justify the use of the FP as a conceptual model. Many studies use food diaries to monitor nutritional status, but this would be difficult in this setting, given the low literacy of our studied population. Frailty is considered an important non-AIDS condition in HIV-infected individuals [35]. One weakness of this study is that HIV status was not tested, rather we relied on self-disclosure. Consequently, we may have underestimated the prevalence of HIV infection as only four participants disclosed their HIV-infection status. HIV prevalence in our background population is unknown but likely to be low based on anonymised testing conducted for a stroke risk factors study in Hai district between 2003 and 6 [36], yet this may limit the generalisability of our findings to other regions of SSA with a higher background prevalence of HIV infection. For example the recent Agincourt frailty study, reported that 21% aged ≥40 years were known to be HIV-infected [6].

A limitation of using the FP to identify frailty in this context, is its focus on physical frailty, excluding other domains of assessment, such as social and economic domains. Particularly in this rural SSA setting, older adults’ frailty status, as well as the outcomes of frailty, may be closely associated with their social and economic resources. Further study is required in order to investigate the impact of these domains.

### Study strengths

This study adds substantially to the limited existing body of knowledge on frailty in older rural-dwelling Africans. The FP has been successfully applied across language and cultural barriers to produce a meaningful measure close to the original phenotypic model. One significant strength of this study is in its hypothesis generation. As a result of our findings of high levels of self-reported weight loss and underweight, we propose that chronic undernutrition due to food insecurity and socio-economic factors may be important in the cyclical deterioration of energy and physiological reserve described by the “cycle of frailty” framework, and deserves further investigation in this setting.

## Conclusion

The study demonstrates that the FP can be successfully operationalised in rural Tanzania to estimate frailty prevalence. The clinical application of the FP in rural SSA is unclear. JAMAR dynamometers are expensive and such technical specialist equipment is unlikely to be available for use routinely as a screening tool. Additionally, if the FP were to be applied clinically, referral for further specialist care would not be possible given the regional lack of human resources for geriatric medicine [37]. Further work assessing the nutritional status of older adults in rural SSA would help to characterise the association between chronic undernutrition and frailty in this setting. Additionally, future research should explore the optimal practical application of the FP, particularly whether a task-shifting approach to its delivery could be employed in order to reach the most vulnerable rural-dwelling older Africans.

## Additional file

Additional file 1: **Table S1.** The frailty status and diagnoses of the 39 participants with missing Hai DSS FP data. (DOCX 16 kb)

## Abbreviations

B-FIT: Brief Frailty Instrument for Tanzania
BMI: Body Mass Index
CES-D: Centre for Epidemiological Studies Depression Scale
CGA: Comprehensive Geriatric Assessment
DSS: Demographic Surveillance Site
FP: Frailty Phenotype
HGS: Hand-grip strength
HICs: High-income countries
IADL: Instrumental Activities of Daily Living
IDEA: Identification and Intervention for Dementia in Elderly Africans
IPAQ: International Physical Activity Questionnaire
ODK: Open Data Kit
SSA: sub-Saharan Africa
WHO SAGE: World Health Organization Study of AGEing and adult health

## References

[1] Fried LP, Tangen CM, Walston J, Newman AB, Hirsch C, Gottdiener J, Seeman T, Tracy R, Kop WJ, Burke G (2001). Frailty in older adults: evidence for a phenotype. J Gerontol A Biol Sci Med Sci.

[2] Fried LP, Ferrucci L, Darer J, Williamson JD, Anderson G (2004). Untangling the concepts of disability, frailty, and comorbidity: implications for improved targeting and care. J Gerontol A Biol Sci Med Sci.

[3] Fried LP, Walston J: Frailty and failure to thrive in: *Geriatric Medicine and Gerontology*. Edited by Hazzard WR, Blass JP, Ettinger WH Jr, Halter JB, Ouslander J, 5 edition (1 July 2003) edn New York: McGraw-Hill Medical; 2003: 1487–1502.

[4] Gray WKRJ, McGuire J, Dewhurst F, Elder V, Weeks J, Walker RW, Dotchin CL (2016). Frailty screening in low- and middle-income countries: a systematic review. J Am Geriatr Soc.

[5] Theou O, Cann L, Blodgett J, Wallace LM, Brothers TD, Rockwood K (2015). Modifications to the frailty phenotype criteria: systematic review of the current literature and investigation of 262 frailty phenotypes in the survey of health, ageing, and retirement in Europe. Ageing Res Rev.

[6] Payne CF, Wade A, Kabudula CW, Davies JI, Chang AY, Gomez-Olive FX, Kahn K, Berkman LF, Tollman SM, Salomon JA (2017). Prevalence and correlates of frailty in an older rural African population: findings from the HAALSI cohort study. BMC Geriatr.

[7] Cesari M, Gambassi G, van Kan GA, Vellas B (2014). The frailty phenotype and the frailty index: different instruments for different purposes. Age Ageing.

[8] Theou O, Rockwood K, Theou O, Rockwood K (2015). Comparison and clinical applications of the frailty phenotype and frailty index approaches. Frailty in Aging Biological, Clinical and Social Implications..

[9] Rockwood K, Mitnitski A (2007). Frailty in relation to the accumulation of deficits. J Gerontol A Biol Sci Med Sci.

[10] Searle SD, Mitnitski A, Gahbauer EA, Gill TM, Rockwood K (2008). A standard procedure for creating a frailty index. BMC Geriatr.

[11] Gray WK, Orega G, Kisoli A, Rogathi J, Paddick SM, Longdon AR, Walker RW, Dewhurst F, Dewhurst M, Chaote P (2017). Identifying frailty and its outcomes in older people in rural Tanzania. Exp Aging Res.

[12] Lewis EG, Wood G, Howorth K, Shah B, Mulligan L, Kissima J, Dotchin C, Gray W, Urasa S, Walker R. Prevalence of frailty in older community-dwelling Tanzanians according to comprehensive geriatric assessment. J Am Geriatr Soc. 2018. 10.1111/jgs.15433 29897098

[13] Orme JG, Reis J, Herz EJ (1986). Factorial and discriminant validity of the Center for Epidemiological Studies Depression (CES-D) scale. J Clin Psychol.

[14] Craig CL, Marshall AL, Sjostrom M, Bauman AE, Booth ML, Ainsworth BE, Pratt M, Ekelund U, Yngve A, Sallis JF (2003). International physical activity questionnaire: 12-country reliability and validity. Med Sci Sports Exerc.

[15] Gureje O, Oladeji BD, Abiona T, Chatterji S (2014). Profile and determinants of successful aging in the Ibadan study of ageing. J Am Geriatr Soc.

[16] Collingwood CPS-M, Kisoli A, Dotchin CL, Gray WK, Mbowe G, Mkenda S, Urasa S, Mushi D, Chaote P (2014). Development and community-based validation of the IDEA study instrumental activities of daily living (IDEA-IADL) questionnaire. Glob Health Action.

[17] Paddick SM, Gray WK, Ogunjimi L, Lwezuala B, Olakehinde O, Kisoli A, Kissima J, Mbowe G, Mkenda S, Dotchin CL (2015). Validation of the identification and intervention for dementia in elderly Africans (IDEA) cognitive screen in Nigeria and Tanzania. BMC Geriatr.

[18] Guerra M, Ferri C, Llibre J, Prina AM, Prince M (2015). Psychometric properties of EURO-D, a geriatric depression scale: a cross-cultural validation study. BMC Psychiatry.

[19] Brunette W, Sundt M, Dell N, Chaudhri R, Breit N, Borriello G. Open data kit 2.0: expanding and refining information Services for Developing Regions. In: ACM HotMobile’13; 2013. http://www.hotmobile.org/2013/papers/full/2.pdf.

[20] Ravindrarajah R, Lee DM, Pye SR, Gielen E, Boonen S, Vanderschueren D, Pendleton N, Finn JD, Tajar A, O'Connell MD (2013). The ability of three different models of frailty to predict all-cause mortality: results from the European male aging study (EMAS). Arch Gerontol Geriatr.

[21] Leong DP, Teo KK, Rangarajan S, Kutty VR, Lanas F, Hui C, Quanyong X, Zhenzhen Q, Jinhua T, Noorhassim I (2016). Reference ranges of handgrip strength from 125,462 healthy adults in 21 countries: a prospective urban rural epidemiologic (PURE) study. J Cachexia Sarcopenia Muscle.

[22] Dodds RM, Syddall HE, Cooper R, Kuh D, Cooper C, Sayer AA (2016). Global variation in grip strength: a systematic review and meta-analysis of normative data. Age Ageing.

[23] Koopman JJ, van Bodegom D, van Heemst D, Westendorp RG (2015). Handgrip strength, ageing and mortality in rural Africa. Age Ageing.

[24] Hurst L, Stafford M, Cooper R, Hardy R, Richards M, Kuh D (2013). Lifetime socioeconomic inequalities in physical and cognitive aging. Am J Public Health.

[25] McLean RR, Mangano KM, Hannan MT, Kiel DP, Sahni S (2016). Dietary protein intake is protective against loss of grip strength among older adults in the Framingham offspring cohort. J Gerontol A Biol Sci Med Sci.

[26] Chilima DM, Ismail SJ (2001). Nutrition and handgrip strength of older adults in rural Malawi. Public Health Nutr.

[27] Capistrant BD, Glymour MM, Berkman LF (2014). Assessing mobility difficulties for cross-national comparisons: results from the World Health Organization study on global AGEing and adult health. J Am Geriatr Soc.

[28] Studenski S, Perera S, Patel K, Rosano C, Faulkner K, Inzitari M, Brach J, Chandler J, Cawthon P, Connor EB (2011). Gait speed and survival in older adults. JAMA.

[29] Britainthinks: Frailty: Language and Perceptions A report prepared by BritainThinks on behalf of Age UK and the British Geriatrics Society In*.*; 2015.

[30] Matoti-Mvalo T, Puoane T (2011). Perceptions of body size and its association with HIV/AIDS. S Afr J Clin Nutr.

[31] Renzaho AMN (2004). Fat, rich and beautiful: changing socio-cultural paradigms associated with obesity risk, nutritional status and refugee children from sub-Saharan Africa. Health & Place.

[32] Jésus P, Guerchet M, Pilleron S, Fayemendy P, Maxime Mouanga A, Mbelesso P, Preux PM, Desport JC (2017). Undernutrition and obesity among elderly people living in two cities of developing countries: prevalence and associated factors in the EDAC study. Clin Nutri ESPEN.

[33] Blaney S, Beaudry M, Latham M, Thibault M (2009). Nutritional status and dietary adequacy in rural communities of a protected area in Gabon. Public Health Nutr.

[34] Cheserek MJ, Waudo JN, Tuitoek PJ, Msuya JM, Kikafunda JK (2012). Nutritional vulnerability of older persons living in urban areas of Lake Victoria Basin in East Africa: a cross sectional survey. J Nutr Gerontol Geriatr.

[35] Brothers TD, Kirkland S, Guaraldi G, Falutz J, Theou O, Johnston BL, Rockwood K (2014). Frailty in people aging with human immunodeficiency virus (HIV) infection. J Infect Dis.

[36] Walker RW, Jusabani A, Aris E, Gray WK, Unwin N, Swai M, Alberti G, Mugusi F (2013). Stroke risk factors in an incident population in urban and rural Tanzania: a prospective, community-based, case-control study. Lancet Glob Health.

[37] Dotchin CL, Akinyemi RO, Gray WK, Walker RW (2013). Geriatric medicine: services and training in Africa. Age Ageing.

