# Supplementary material for: The prevalence and characteristics of frailty by frailty phenotype in rural Tanzania
Source: BMC Geriatr. 2018 Nov 16;18:283. doi: 10.1186/s12877-018-0967-0 (PMC6240208; doi:10.1186/s12877-018-0967-0)
Supplement: Supplementary file 1 — Table S1. The frailty status and diagnoses of the 39 participants with missing Hai DSS FP data. (DOCX 16 kb) [file 12877_2018_967_MOESM1_ESM.docx]

Additional file 1: Table S1 The frailty status and diagnoses of the 39 participants with missing Hai DSS FP data

| Age and sex | Frailty status by CGA | Missing FP parameters | Mobility status | Diagnoses |
| --- | --- | --- | --- | --- |
| 63 male | Not frail | Walking speed, (erroneous data deleted) | Walks independently | None |
| 83 male | Not frail | HGS | Walks Independently | OA, BPH Has a wrist brace on the left wrist following a motorbike accident. |
| 85 male | Not frail | HGS | Walks Independently | HTN, OA, TIA, Doesn’t attend church because his joints get stiff from sitting for too long. |
| 68 female | Frail | Walking speed | Wheelchair-Bound | Old polio, depression |
| 71 female | Frail | Walking speed, HGS, Exhaustion | Immobile | Stroke, HTN, |
| 61 male | Frail | Walking speed | Immobile | Old polio, |
| 85 female | Frail | Walking speed, HGS, Exhaustion | Immobile | Dementia |
| 80 male | Frail | Walking speed HGS | Immobile | Malnourished, cataracts, |
| 87 male | Frail | Walking speed HGS | Wheelchair-bound | RA with possible OA, essential tremor, urge urinary incontinence, visual impairment right eye |
| 84 male | Frail | Walking speed | Walks with Assistance | OA, palpitations, ETOH excess, multiple falls |
| 84 male | Frail | Walking speed, HGS | Immobile | HTN, Asthma, stroke, RA |
| 89 female | Frail | Walking speed | Immobile | OA, HTN, chronic cough, incontinence of urine, PUD, cataracts |
| 104 female | Frail | Walking speed | Walks with Assistance | OA, cataracts, GORD, |
| 81 male | Frail | Walking speed, HGS | Immobile | DM Type 2, bilateral AKA for chronic infected foot ulcers, supra-public catheter ? BPH or neuropathy |
| 70 male | Frail | Walking speed, HGS | Immobile | Pituitary macroadenoma, seizures, chronic hyponatraemia, cataracts |
| 90 female | Frail | Walking speed, HGS | Immobile | Stroke, HTN, contractures, |
| 103 female | Frail | Walking speed | Immobile | PUD, Asthma, OA |
| 80 male | Frail | Walking speed, HGS | Immobile | HTN, Stroke, urinary incontinence catheterised |
| 75 female | Frail | Walking speed | Immobile | Asthma/COPD, HTN, Bilateral arthritis small joints of the hands |
| 91 male | Frail | Walking speed | Walks with Assistance | visual impairment, PUD |
| 79 male | Frail | Walking speed, HGS | Immobile | HTN, Stroke, chronic cough, bed sores, |
| 75 male | Frail | Walking speed | Walks Independently | Cognitive impairment, cataracts |
| 93 female | Frail | Walking speed, HGS | Immobile | Stroke, Urinary incontinence, HTN |
| 81 male | Frail | Walking speed | Immobile | Left AKA for gangrene, PVD |
| 93 female | Frail | Walking speed | Immobile | Dementia with visual hallucinations |
| 90 female | Frail | Walking speed | Immobile | HTN, DM Type 2, Stroke |
| 95 female | Frail | Walking speed, HGS | Immobile | Dementia, OA |
| 88 female | Frail | Walking speed | Immobile | Osteoporosis, OA, disc problems |
| 82 female | Frail | Walking speed | Immobile | OA, HIV, chronic cough |
| 75 female | Frail | Walking speed | Immobile | HTN, Stroke |
| 90 female | Frail | Walking speed | Immobile | Lower limb paralysis ? TB of spine, urinary incontinence, constipation, chronic wheeze ? Post TB bronchiectasis ? COPD |
| 110 female | Frail | Walking speed, HGS | Immobile | Stroke, probable aspiration pneumonia, HTN, pressure sores, contractures |
| 62 male | Frail | Walking speed, HGS | Immobile | Stroke, urinary incontinence, dementia, HTN, pedal oedema |
| 104 female | Frail | Walking speed | Immobile | Cognitive impairment, Visual impairment due to bilateral cataracts, immobility |
| 90 female | Frail | Walking speed (erroneous data deleted) | Walks with Assistance | Visual impairment, goitre, ? COPD, OA |
| 87 female | Frail | Walking speed, HGS | Immobile | Visual impairment, bowel and bladder incontinence |
| 79 female | Frail | Walking speed | Walks with Assistance | COPD, haemorrhoids, #NOF following fall-not operated, lipoma, urinary and faecal incontinence, cataracts |
| 77 female | Frail | Walking speed, HGS | Walks Independently | Dementia, visual impairment, OA |
| 68 female | Frail | Walking speed | Wheelchair | Old polio, depression |
